# Blockchain in manufacturing quality control: A computer simulation study

**DOI:** 10.1371/journal.pone.0247925

**Published:** 2021-03-01

**Authors:** Pooi-Mun Wong, Shreya R. K. Sinha, Chee-Kong Chui

**Affiliations:** Department of Mechanical Engineering, National University of Singapore, Singapore, Singapore; National Textile University, PAKISTAN

## Abstract

Blockchain has been applied to quality control in manufacturing, but the problems of false defect detections and lack of data transparency remain. This paper proposes a framework, Blockchain Quality Controller (BCQC), to overcome these limitations while fortifying data security. BCQC utilizes blockchain and Internet-of-Things to form a peer-to-peer supervision network. This paper also proposes a consensus algorithm, Quality Defect Tolerance (QDT), to adopt blockchain for during-production quality control. Simulation results show that BCQC enhances data security and improves defect detections. Although the time taken for the quality control process increases with the number of nodes in blockchain, the application of QDT allows multiple inspections on a workpiece to be consolidated at a faster pace, effectively speeding up the entire quality control process. The BCQC and QDT can improve the quality of parts produced for mass personalization manufacturing.

## Introduction

In 2008, Satoshi Nakamoto popularized blockchain by introducing a decentralized system that validates transactions without depending on a third party [1]. Blockchain is a growing ledger that records encrypted pools of transaction data into a block. Each block is linked to its previous block, and thus, forming a chain. This growing ledger is immutable by design. Although blockchain was originally used in finance-related applications, it has been extended to non-finance applications, such as internet-of-things (IoT) and healthcare [2]. Its application in manufacturing improves real-time transparency, reduces cost, and assures sustainability [3].

During-production quality control (QC) of a workpiece refers to verification and validation of the workpiece while it is still in production. Conventional manufacturing typically performs QC in a centralized manner and obtains inspection results in a one-time manner. Workpieces are randomly sampled for inspection because inspecting individual workpieces is often costlier than the production cost [4, 5]. However, there are various potential problems with this method. A potential problem is the occurrence of human error during manual inspections which contributes to false defect detections [6]. Such human error may be due to systematic error or random error. If the cause is random error, the false defect detections can be reduced through repetitive workpiece inspection. Another potential problem is the complication of responsibility tracing within QC processes due to erroneous amendment of inspection data. Besides that, the presence of materials-at-risk and late defect detections could result in scrapping or reworking the workpiece [5]. This could squander material and manpower. Therefore, the motivation of this paper is to address these issues.

Manufacturing process can be adjusted when real-time monitoring detects defects in workpieces [7]. Interconnected sensors in IoT could realize such real-time monitoring. The IoT could be coupled with the adoption of blockchain to provide data security among sensors [2]. Ho et al. [8] have shown that the use of blockchain greatly improves the quality of parts produced in a cyber-physical system (CPS). This improvement was due to multiple validation checks from other nodes in the network. Hence, this paper expands on the concept of [8] by building upon the current advances in blockchain and IoT. We envision that blockchain in manufacturing has practical applications in the mass production of high-value personalized products, such as medical implants.

The objective of this research is to investigate the feasibility of decentralizing the responsibility of QC into a peer-to-peer supervision network. Simultaneously, this research attempts to ensure that this responsibility is secure and traceable. The paper proposes a blockchain framework, named Blockchain Quality Controller (BCQC), and a consensus algorithm, named Quality Defect Tolerance (QDT). BCQC involves peer-to-peer supervision and records the finalized inspection results. To be specific, the blockchain controls the supervision and acts as a distributed ledger during production. Within BCQC, each blockchain node contributes an inspection result. Similar to other blockchain models, the BCQC needs a consensus algorithm to realize a decentralized and trustless network. However, existing consensus algorithms are not suitable for QC purposes due to two reasons: (1) their focus is on ensuring that transaction data are untampered, and (2) allocation of computational resources focuses mainly on the selection of a worthy node to create a block. Since a decentralized QC system may have multiple inspection results for a single workpiece, the focus of consensus should instead be on selecting trustworthy inspection results. This focus should be prioritized even if the data are untampered. In such cases, the selection of a worthy node to create a block loses its priority over the consolidation of inspection results.

This motivates the development of our novel consensus algorithm, QDT, which is applicable in BCQC or in general QC systems. The main goal of QDT is to select an inspection result from a pool of inspection results that is collated from multiple blockchain nodes. To evaluate the effectiveness of BCQC and QDT, the following simulations are performed: (1) production-dependent QC with multiple inspectors, (2) production-independent QC with QDT, and (3) production-dependent QC with QDT. The parameters studied are F1 score of defect detection and total manufacturing time (which include production time and QC time).

This paper hypothesizes that the BCQC framework could improve the chances of detecting a defective workpiece at an early stage. This improvement is achieved by having multiple inspectors inspect the workpiece after each manufacturing step. These inspections may be performed either on the physical workpiece or on the data collected by a single sensor. This paper also hypothesizes that the QDT algorithm could improve the transparency of inspection results. Although all inspection results may not be the same, QDT will consolidate the multiple results into one to be added to the blockchain. The blockchain will then be updated for the whole production line, thereby ensuring transparency of the inspection results.

The paper is organized as follows. Section 2 explains the mechanism of blockchain. Section 3 presents literature related to QC in manufacturing and blockchain in process control. Section 4 describes the proposed BCQC and QDT. Section 5 details computer simulations and evaluation results of BCQC and QDT in various scenarios. The last section concludes the paper and discusses potential future work.

## Blockchain fundamentals [1, 9]

A node is a user or device that can mine or verify blocks in the blockchain. A node can be either a miner or a non-miner. A miner is selected based on a consensus algorithm from a pool of miner nodes. The miner applies a hash function (such as SHA-256 and Blake-256) to encrypt data of arbitrary size (typically of type String) to data of a fixed size. In most finance applications, this encrypted data is a pool of transactions. The data of fixed size is known as hash values. The decryption of the hash value is relatively easy compared to the process of encrypting the data into the hash value. In reference to the name of the mechanism, each hash value represents a block. Each block is validated by the other nodes before being accepted into the chain. The blocks are chained together by including the hash values of the previous block into the unencrypted data of each block.

Bitcoin is one of the earliest commercial implementations of such blockchain technology. Proof of work (PoW) is a consensus algorithm used in Bitcoin where only hash values with a predefined prefix will be accepted to be added to the blockchain. This means that the hash of the data has to begin with that prefix. Hence, some arbitrary characters, known as a nonce, is added to the data to achieve that hash value. Selection of the nonce is done through a trial and error process and is computationally expensive. There are other existing consensus algorithms, for example, proof of stake (PoS) that is used in Ethereum and practical byzantine fault tolerance (PBFT) that is used in Hyperledge; both are explained in [10].

Ethereum is a blockchain technology that offers greater processing capability and allows programming codes written in different programming languages to run within the chain. A piece of programming code is known as a smart contract. Once hashed into the block, the smart contract has no owner and its content cannot be tampered. Despite this additional capability, Ethereum is mostly used for cryptocurrency transactions. To date, only very few simplistic smart contracts can be found within Ethereum [11].

## Related work

A review paper on assembly systems for Industry 4.0 [12] reported numerous advancements in smart sensors, gauges, cameras, and documentation technologies that support quality control and management. Blockchain was one of the technologies that were expected to bring extensive benefits to the assembly system.

In manufacturing, especially in the context of Industry 4.0, blockchain was often proposed for data management as a distributed ledger [10, 13–15]. Besides that, blockchain may facilitate cloud manufacturing based on its trustless communication network [16]. In supply chains, blockchain has been proposed in data management for product flow traceability and financial intermediaries [17]. Lu and Xu [18] reports the deployment of blockchain for product flow tracing where each product has a dedicated blockchain that stores the ownership and product status. Similarly, the application of blockchain in quality control has been investigated for the supply chain management. However, it was not applied at the production level [19]. Blockchain has also been proposed as a trustless system to enable shared manufacturing of a product [20, 21].

With the development of Industry 4.0 in mind, technologies such as the IoT, CPS, and wireless sensor networks (WSN) encourage the installation of sensors and smart machines within the production line. This has resulted in sensors and equipment becoming increasingly accessible. Yin et al. [22] anticipated that the openness of such Industry 4.0 technologies will make manufacturing systems more vulnerable to cyberattacks. Hence, enhanced security on quality control results might be needed and could be achieved using blockchain. Furthermore, the trustless nature of blockchain could reduce the workload of supervisors in the production line while producing reliable results. However, Rakovic et al. [10] reported that the current consensus algorithm is not suitable for IoT due to the high computational power and lengthy decision processes. IoT involves massive data exchange in which storage could become very costly, and hence it is important to design on-chain data by considering performance and data privacy [18]. Casino et al. [23] proposed that applications beyond cryptocurrency and smart contracts should be termed Blockchain 3.0, and presented a systematic review of the suitability of blockchain in IoT.

Blockchain utilizes proof-based or/and vote-based consensus algorithm. In proof-based algorithms, multiple miners may exist but only the results from the best miner are accepted [24]. Occasionally, this may lead to computationally resourceful miners overpowering the other nodes. This issue is observed in PoW where only a few resourceful nodes hold authoritative power without penalty [25]. In spite of this, new proof-based algorithms are still being proposed in the literature. One example is the proof of participation (PoP) that was proposed for shared manufacturing to record data of shared resources [21]. Each node carried a score of participation level. If the score exceeded the expected value, the node was determined to be eligible to mine the block. Conflicts were resolved by accepting the chain from the miner with a higher participation level. This algorithm is similar to PoS where the nodes with high cumulated stakes are eligible to mine the block.

On the other hand, in vote-based algorithms, only one miner exists and is selected based on votes [24]. This method eliminates the possibilities of resourceful nodes monopolizing the miner position but is highly influenced by the trustworthiness of voters. To counter the influence of lowly trustworthy voters, vote-based algorithms could be strengthened by introducing weighted voting based on the voter’s profile [26]. Lastly, there is a consensus algorithm that combines proof-based and vote-based algorithms. This algorithm is PBFT and allows a node to mine for many rounds until it is found to exhibit anomalous behaviours. However, compared to vote-based algorithm, PBFT is less scalable as the rise in the number of nodes may result in high overhead cost and a lower likelihood of reaching consensus [19].

Leveraging the decentralized characteristic of blockchain for trustless peer-to-peer supervision in product manufacturing should be explored. It is important to keep the limitations of existing blockchain algorithms in mind when designing a QC system for manufacturing. These limitations are comprehensively reviewed by [27]. Limitations such as low throughput and high latency can be largely attributed to the consensus algorithm of the blockchain. Furthermore, there may be security threats when blockchain is used for fast transactions.

In product manufacturing, apart from data management, the QC process involves inspection assignment, verification, and validation of the physical workpiece. As the current blockchain framework only focuses on data management and smart contracts, features for verification and validation of physical workpieces are lacking. This limitation becomes an obstacle in adopting blockchain for the QC process. Hence, this paper explores the possibility of incorporating these features into a blockchain network.

## Methods and materials

### Blockchain framework

**Fig 1:**
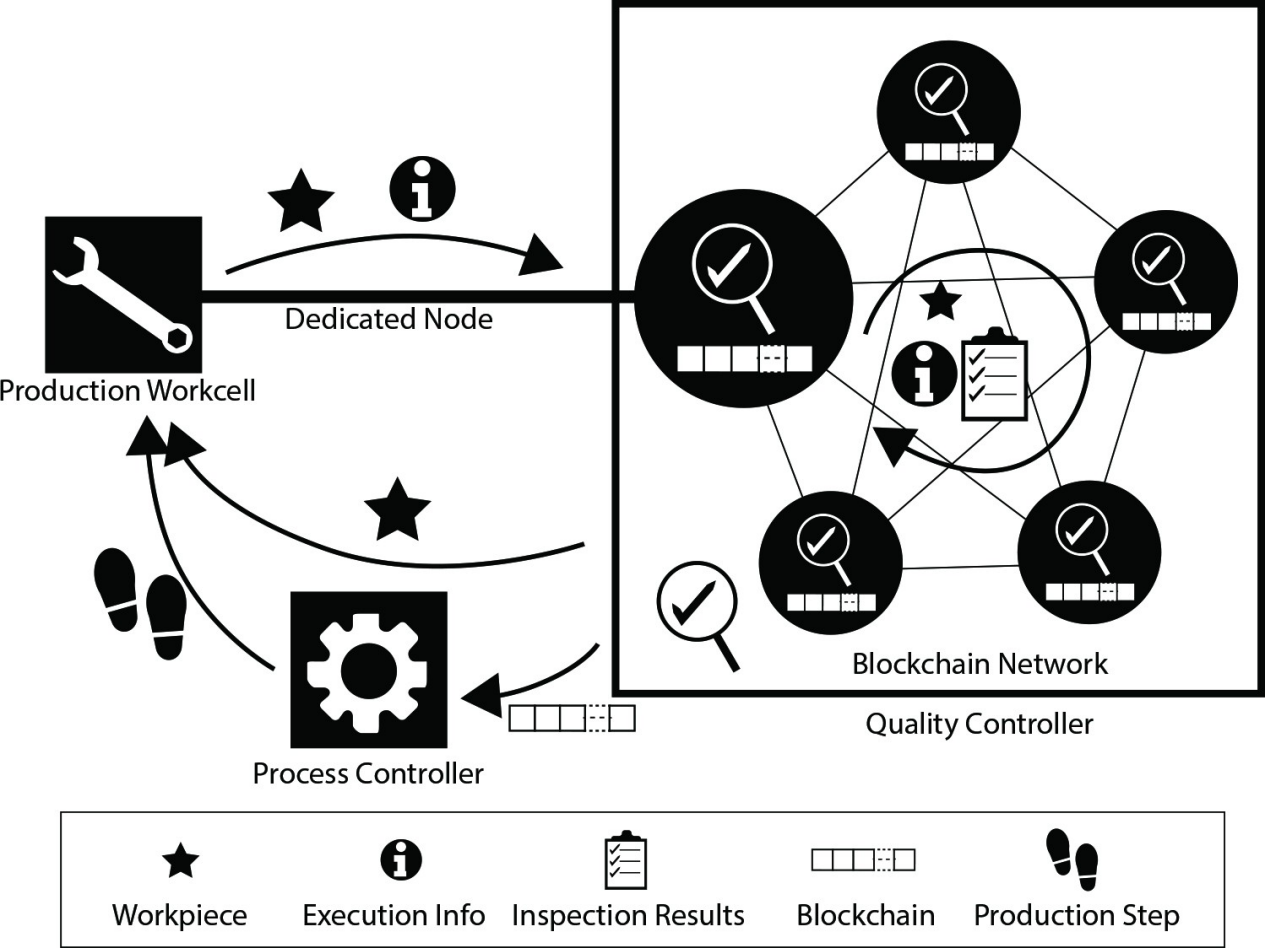
High-level architecture of BCQC.

Fig 1 illustrates the key components involved in the proposed QC process. A production workcell is a CPS unit that performs tasks on the workpiece. Among the production workcells, each workcell that performs QC has a dedicated node in a blockchain network. When the production workcell has completed a production step, it passes the workpiece with its execution information to its dedicated node. This action initiates the QC process. The dedicated node performs the first inspection on the workpiece and then passes the workpiece into the blockchain network that comprises multiple nodes. These nodes take turns to perform the necessary inspections and conclude the QC cycle after a consensus is reached. Next, the inspection results and production information that are consolidated in the achieved consensus are recorded into a blockchain.

Each blockchain represents a workpiece. When the workpiece is found to be defective, information about its defects is also recorded into the blockchain. Although the defective workpiece is scrapped, information about its defect may be analyzed to identify failure points and perform prognosis on machine health for system improvement. After this information is analyzed, its respective blockchain could be destroyed.

Subsequently, the recorded data are sent to the process controller to decide on the next production step. In view of multiple personalized production (MPP) where alternative production sequences are possible, flexibility in this decision-making process is encouraged. Such flexibility could be promoted via the transference of prior information that provides insights into the entire situation. By using blockchain, the transferred information cannot be tampered with, enabling reliable data tracking and error tracing. Once the decision on the next step is made, the workpiece will be sent to a workcell to perform that step.

Within each QC cycle, it is assumed that each node (inspector) has a certain capability for inspection. Estimation of this capability can be represented mathematically by a score. The score is used to compute the confidence level of an inspection result that is given by various nodes. Inspection result is a list of states that describes the workpiece. This is similar to a quality control check sheet. Taking the inspection of a hole to be drilled as an example, the parameter to be inspected is its diameter, with the desired value of 5 mm with tolerance +/- 0.2 mm. Three possible states for this inspection could be defined—Small for diameter below 0.48 mm, Fit for diameter within 0.48 mm to 0.52 mm, and Large for diameter above 0.52 mm. These inspection results are packed into a transaction. At the same time, information of the assembly step and the group of nodes that contributed to that results are also included in the transaction. Then, the hash of the immediate previous block is added to the transaction as a header for the block.

### Blockchain quality controller (BCQC)

**Fig 2:**
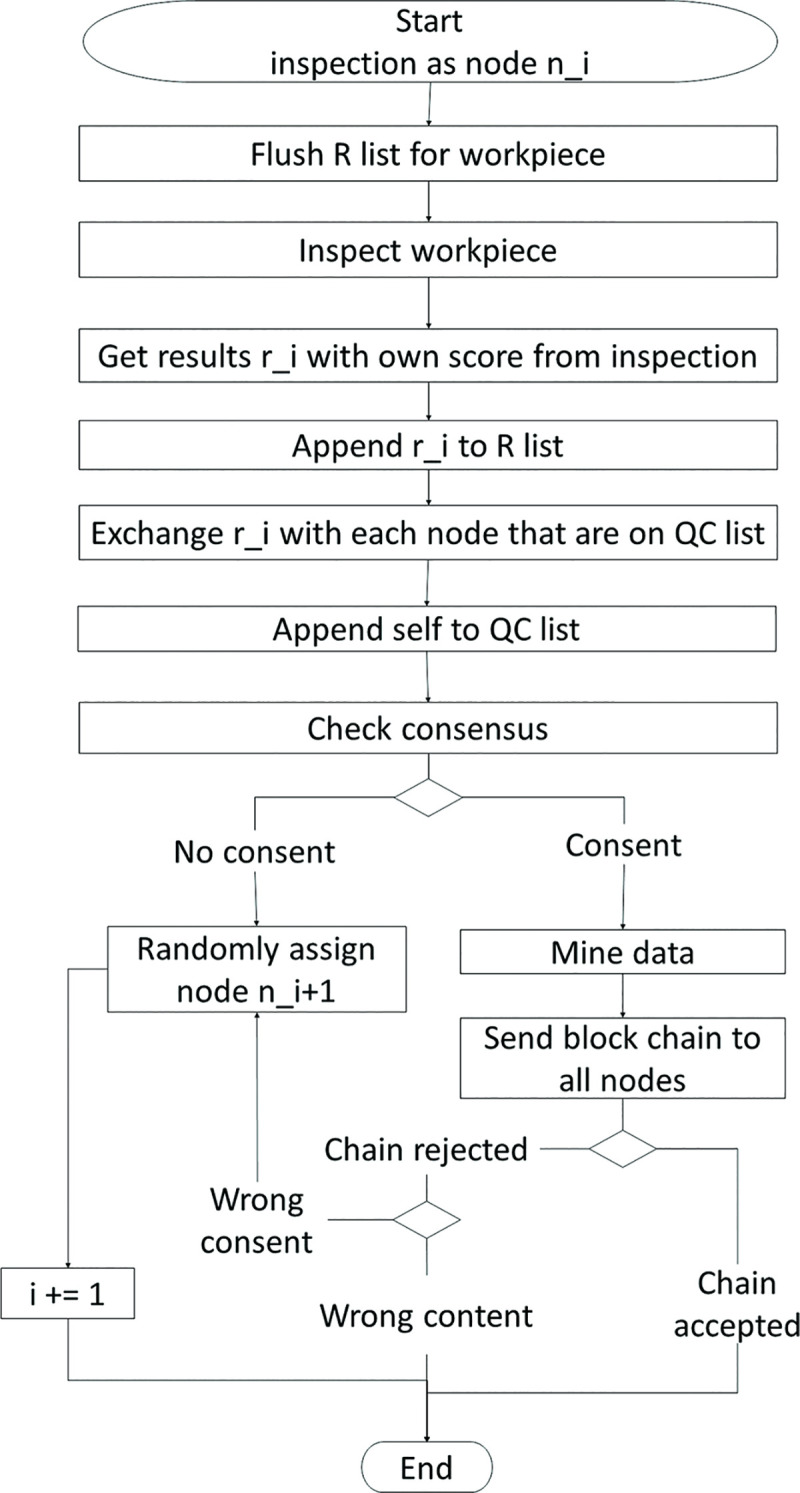
Flow chart for a blockchain node being involved in workpiece inspection.

**Fig 3:**
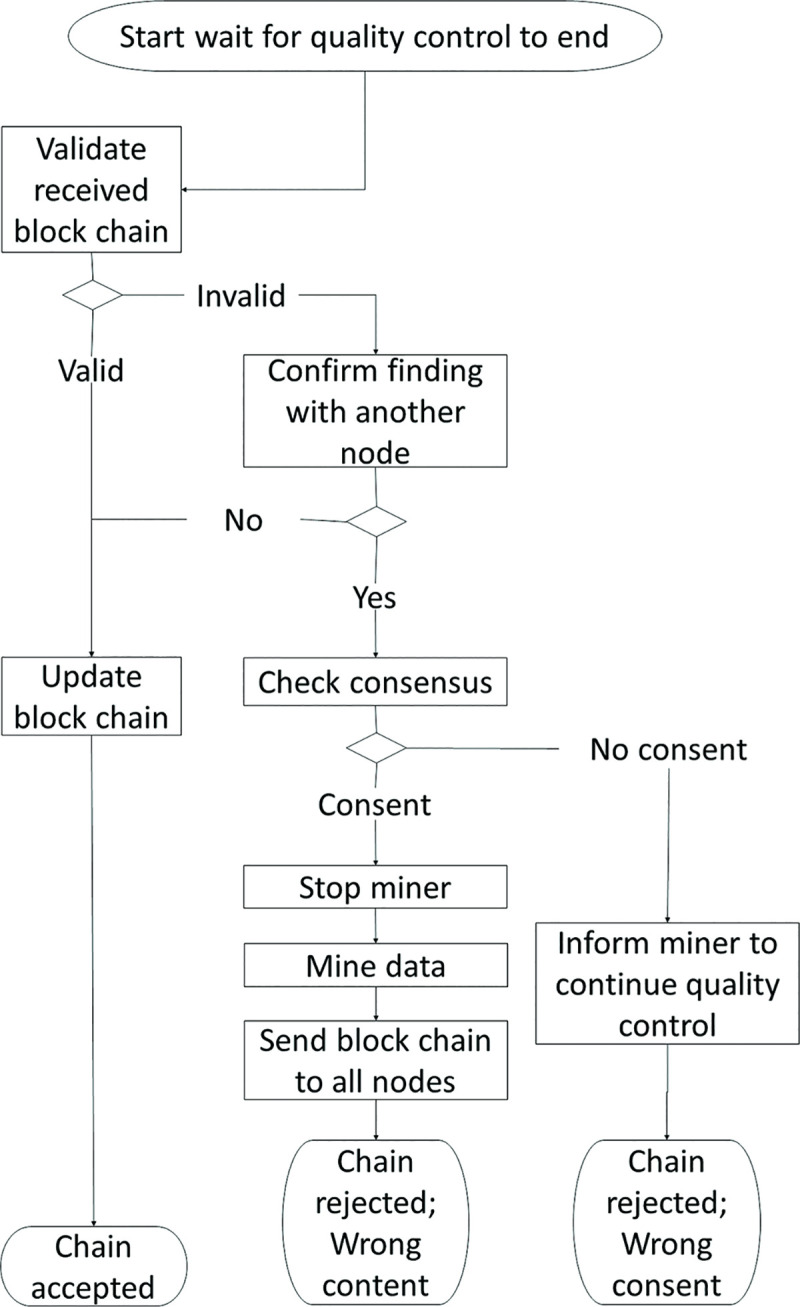
Flow chart for a blockchain node not involved in workpiece inspection.

Figs 2 and 3 describe the process of BCQC performed on a workpiece. Upon completion of a production step, the production workcell assigns its dedicated node, *n*_0_, to inspect the workpiece. Let *n*_*i*_ be the i-th node on the QC list. The workcell first assigns *n*_0_ to start the inspection and appends *n*_1_ into the QC list. Three items will be passed from node to node: (1) the QC list, (2) information of the execution, and (3) information of the workpiece. Every node has a private cache that is not shared with the other nodes. This private cache stores its own inspection results of each workpiece.

After *n*_1_ has completed its inspection, *n*_1_ has to select another node to inspect the workpiece. Making the selection in a fixed sequential order may induce a bias in the inspection results. For example, scheduling a prone-to-error Inspector B after Inspector A may result in the following: (1) The inspections will be consistently unreliable due to erroneous judgement by Inspector B. This leads to requiring more inspectors to achieve a consensus on the result; (2) Error identification will be more challenging. It will be difficult to identify the source of error among the inspectors. With the above in mind and to reduce the mentioned bias, the next node is selected at random since the nodes in the blockchain are interconnected. Furthermore, this randomness cross-checks the performance of each inspector. For example, the frequent occurrence of errors whenever Inspector B takes part in the inspection step would suggest Inspector B as a possible source of error. Hence, *n*_1_ will randomly select another neighboring node that has sufficient capability, *n*_2_, to inspect the workpiece.

With that, node *n*_1_ appends *n*_2_ into the QC list. Subsequently, node *n*_1_ transfers the three items to *n*_2_. After *n*_2_ has completed its inspection, *n*_2_ sends its inspection results to the nodes in the QC list. Upon receiving these new results, the nodes return their results to *n*_2_. Thus, the inspection results are disseminated in a one-to-one information exchange method. This method ensures that the data can be stored in a distributed and redundant manner. This method also better ensures data integrity even if data corruption on a node happens. Up to this point in the process, the nodes only store and exchange raw information of the inspection results. Next, consolidation of the results is carried out in a consensus algorithm. If consensus is not met, then *n*_2_ randomly selects another neighboring node that has sufficient capability, *n*_3_, to inspect the workpiece and the process repeats itself in a loop.

When consensus is met, the chain with the newly mined block will be sent to all nodes. Upon receiving the chain, the node will validate all blocks in the chain. If the chain is valid, its current chain will be replaced by this new chain. If the chain is invalid, the node will validate its findings with the other nodes. If consensus is met but the chain is rejected by at least 51% of the nodes on QC list, the node will stop the miner from sending the chain to the rest of the nodes. Subsequently, the process continues in the direction based on the reason for the rejection of the chain. As shown in Fig 3, the loop will continue if an anomaly is found in the calculation of the consensus. If there is a corruption of the content in the last block, one of the nodes that discovered the corruption will replace the miner. The loop ends when consensus is met and the chain is accepted by all nodes.

Inspection can be performed either physically or virtually. Physical inspection is performed by taking measurements on the workpiece. Note that the transportation of the workpiece from one workcell to another will require time depending on the design of the shopfloor. Thus, the distance between workcells and the transportation method has to be taken into account. Virtual inspection is performed by (1) retrieving data of the workpiece from the dedicated node of the production workcell, *n*_0_ and (2) feeding the data to multiple programs that reside in the other nodes to generate evaluation results. The program used across the nodes could be of different deep learning models or of similar architectures but with different weights.

### Quality defect tolerance (QDT)

**Fig 4:**
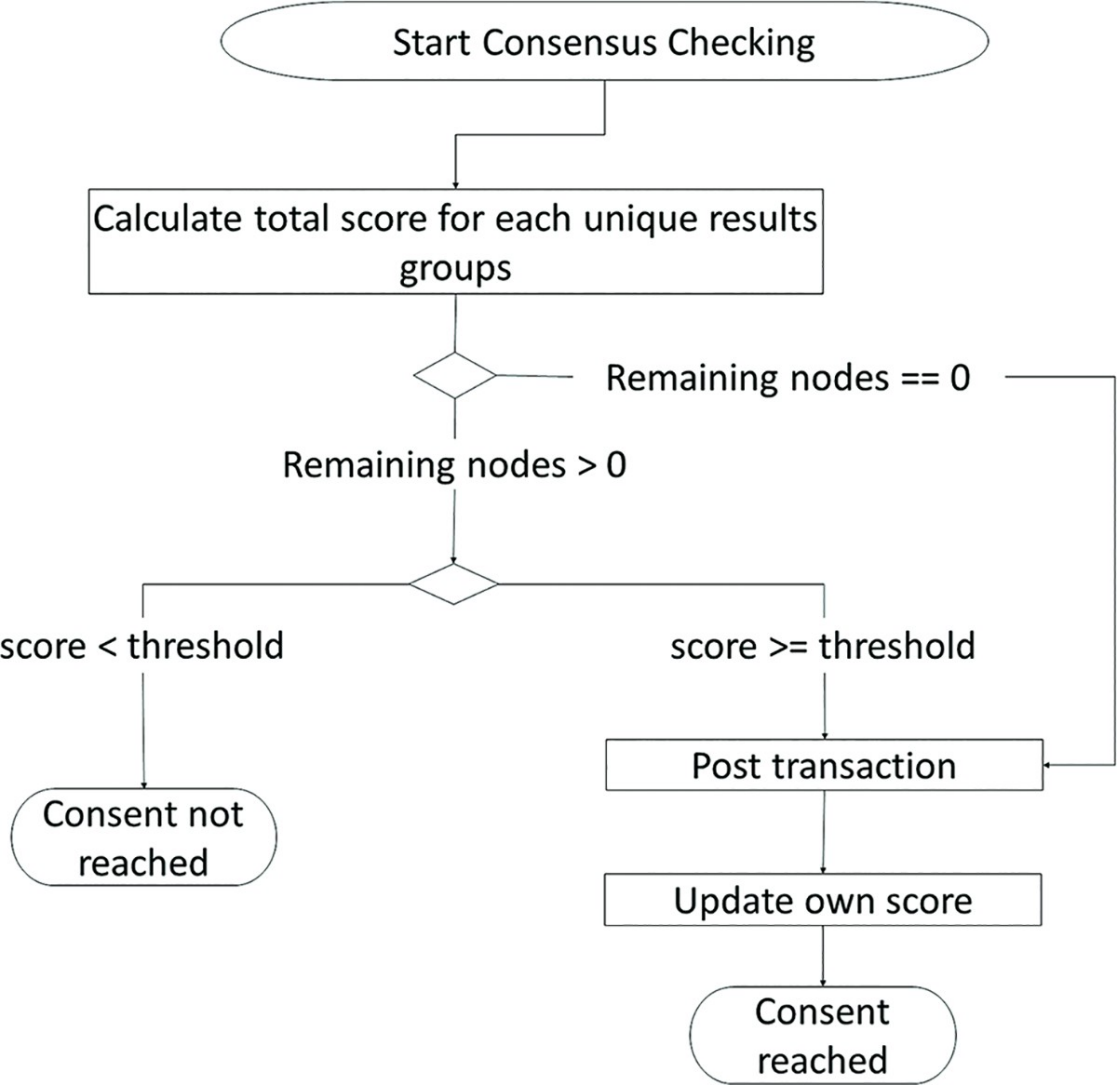
Flow chart for a blockchain node checking for consensus in inspection results.

Fig 4 describes the algorithm of QDT. After a node completes its inspection, the nodes categorize similar results into groups and calculate the accuracy for each results group. Consensus is achieved when either (1) the QC list contains a results group with a confidence level that exceeds a threshold of acceptance or (2) the QC list reaches the maximum allowable number of nodes. In the case of (1), the nodes that contributed to that results group will be labelled as the winner group. In the case of (2), the nodes that contributed to the results group with the highest confidence level will be labelled as the winner group.

Note that the number of minimum allowable nodes must be larger than the possible combinations of states which is described by Eq 1 and has to be rounded up to an odd number:

$$node_{min}=\prod_{i=1}^{N_{state\;categories}}{{n_{states}}_{C}}_{1}.$$ (1)

The consensus conditions mentioned above will affect the required number of inspections. A reduced number of inspections is beneficial in a physical inspection process because workpiece transportation will be reduced. Additionally, competition between multiple miners may not be necessary because the factory can control the distribution of computational load across workstations. Without the competition, the last node from the winner group has the privilege to be a miner upon achieving consensus. Execution information, the inspection results, and information of the winner group that are consolidated in the achieved consensus are packed and mined into a block using a hash function. Then, the block is added to the blockchain.

## Results and discussions

### Desktop computer assembly process

The assembly of a built-to-order desktop computer is used as an example of mass personalization production. Computer simulations were performed in a Python environment using Flask RestAPI as the communication channel between nodes. Python codes for blockchain are modified from [28]. The simulated scenarios focused on an assembly line of Optiplex 9020, which is a desktop model from DELL. The assembly process is designed based on its official user manual. The time taken to assemble each part was obtained from recording the assembly process performed by a novice subject, ranging between 50 to 80 seconds each. The time taken to inspect the workpiece is assumed to be shorter, that is between 20 to 40 seconds. In the simulation, the time taken for these processes was randomly selected from these ranges.

Each part of the desktop computer is given a unique identifier (UID) that is related to their production batches for material tracking purposes. Either a human or an AI agent can inspect the workpiece. While a human cannot perform an assembly step and inspection at the same time, an AI agent is not limited by this restriction and can perform both tasks simultaneously. This affects the time taken of the process and the occurrence of bottlenecks in the assembly line. There are only two possible states for each step: “pass” and “fail”. If the workpiece has successfully reached the end of the manufacturing line with unacceptable quality, the workpiece will be scrapped as waste.

**Fig 5:**
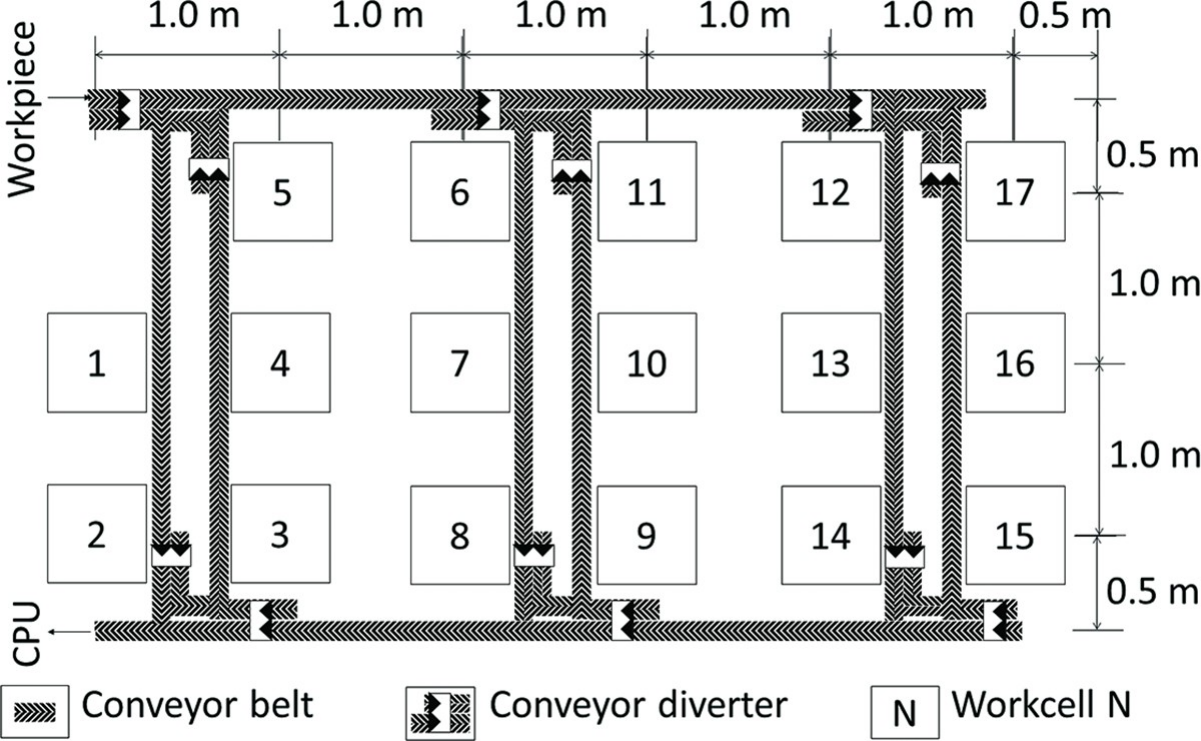
Floor plan of assembly shopfloor.

The performed simulations represent production lines that assemble five products simultaneously. Each assembly process consists of 17 assembly steps as shown in Table 1. Each step is executable by a specific workcell. For ease of reference, each workcell is labelled with the number of the step it can execute, with the layout of the shopfloor shown in Fig 5. All workcells are assumed to have equal capability in inspecting every assembly step. If the workcell is busy with an operation on one workpiece, the workcell is not allowed to work on another workpiece. The workcells are distanced at 1 m apart from the adjacent workcells, and have conveyor belts transporting workpieces between them. With programmable conveyor diverters guiding each workpiece to its targeted workcell, each workcell is accessible from all other workcells. Furthermore, instead of the typical process of random sampling, every workpiece undergoes inspection. This evaluates the efficiency of the algorithm in an extreme scenario, owing to the inevitably longer process for inspecting all workpieces.

**Table 1:** Preconditions of each step in desktop computer assembly.

No.	Step	Preconditions
1	Insert system board.	None.
2	Insert IO panel.	Complete Step 1.
3	Insert power switch.	Complete Step 1.
4	Insert thermal sensor.	Complete Step 1.
5	Insert system fan.	Complete Step 2, 3, and 4.
6	Insert processor.	Complete Step 5.
7	Insert heat sink assembly.	Complete Step 6.
8	Insert power cable.	Complete Step 6.
9	Insert optical drive.	Complete Step 6.
10	Insert hard drive.	Complete Step 6.
11	Insert coin cell battery.	Complete Step 6.
12	Insert memory card.	Complete Step 7, 8, 9, 10, and 11.
13	Insert expansion card.	Complete Step 12.
14	Insert front bezel.	Complete Step 13.
15	Insert WLAN card.	Complete Step 13.
16	Insert intrusion switch.	Complete Step 13.
17	Insert chassis cover.	Complete Step 14, 15, and 16.

The simulations were evaluated based on the time involved in completing the workpieces and the QC F1 score. To generate this confusion matrix that is shown in Table 2, the actual quality of the workpiece for each step was randomly generated. Based on the confusion matrix, F1 score describes the harmonic mean of precision and recall, which emphasizes positive detections of the defective products rather than negative detections. This is especially desirable in manufacturing scenarios, where True Positives (TP), False Positives (FP), and False Negatives (FN) are considered costlier than True Negatives (TN). This higher cost is due to wastage that is produced from scrapping or reworking the workpieces. To control the consistency of the effect of inspection results (pass/fail) from the QC processes on the selection of the next production step, two conditions were designed: (1) “production-dependent”, which means the QC processes will affect the production sequence, and (2) “production-independent”, which means the QC processes will not affect the production sequence.

**Table 2:** Confusion matrix for defective workpiece detection.

	Defective	Not Defective
**Fail**	True positive (TP)	False positive (FP)
**Pass**	False negative (FN)	True negative (TN)

### Production-dependent QC with PoW

The focus of this simulation is to observe the effect of multiple inspections per workpiece on the manufacturing time and workpiece quality. Hence, PoW was used to achieve consensus among the nodes but was limited to only one miner at a time. In this simulation, virtual inspections are performed based on timestamp, part UID, and the pass/fail state output from the executed step. Hence, the physical workpieces are not transported around for inspection and all inspections could be performed at the same time.

It is also assumed that the transportation of the workpiece between production workcells is instantaneous, and hence, the resulting manufacturing time only consists of the time taken to assemble the parts and the time taken to inspect the workpiece. The inspection results that are consolidated in the achieved consensus are supported by more than eight workcells (>50%).

A Conventional QC framework was designated to act as a baseline comparison to the BCQC framework. In this Conventional QC, the workcell that executes the assembly step will inspect its own completed step and will not inspect other steps from the other workcells. There is imperfect communication within the production line. Hence, only some workcells have access to the status of specific production steps. These workcells are defined as “checkpoints” where the workpieces can be reworked if found defective. The checkpoints are (1) Workcell 5, which has access to Step 1 to 5, (2) Workcell 12, which has access to Step 6 to 12, and (3) Workcell 17, which has access to Step 13 to 17.

Conversely, in BCQC, each workcell is a node that can inspect and mine data. The inspectors use fuzzy logic to determine the pass/fail of each step. When consensus is achieved, execution information and the inspection results from the consensus are mined into a block and then added to the blockchain. Each workcell has complete visibility of every step taken that is related to the workpiece. This visibility is due to the transparency of the blockchain. Since there is perfect communication within the production line, all workcells are aware of the status of each other. Thus, the workpiece can be reworked at any and every workcell.

The type of step sequencing was also investigated, i.e. when assembly steps must be executed in serial or can be executed in parallel. In Serial Step Sequence, processes have to be carried out in the predefined chronological order. This can cause bottlenecks when the next production workcell is occupied with another workpiece. In Parallel Step Sequence, assembly steps can be dynamically chosen by a process controller based on preconditions for each step that is listed in Table 2. This allows assemblies to choose available workcells which will reduce the occurrence of bottlenecks. Conventional QC is inherently unable to support Parallel Step Sequence due to its imperfect communication network.

Various scenarios, as described in Table 3, are simulated based on the following parameters: step sequencing method, the number of human inspectors, the number of AI inspectors, and the framework.

**Table 3:** Description of various scenarios.

No.	Step sequence	Human inspector	AI inspector	Framework
1	Serial	9	8	Conventional QC
2	Serial	0	17	BCQC
3	Serial	9	8	BCQC
4	Serial	17	0	BCQC
5	Parallel	0	17	BCQC
6	Parallel	9	8	BCQC
7	Parallel	17	0	BCQC

**Fig 6:**
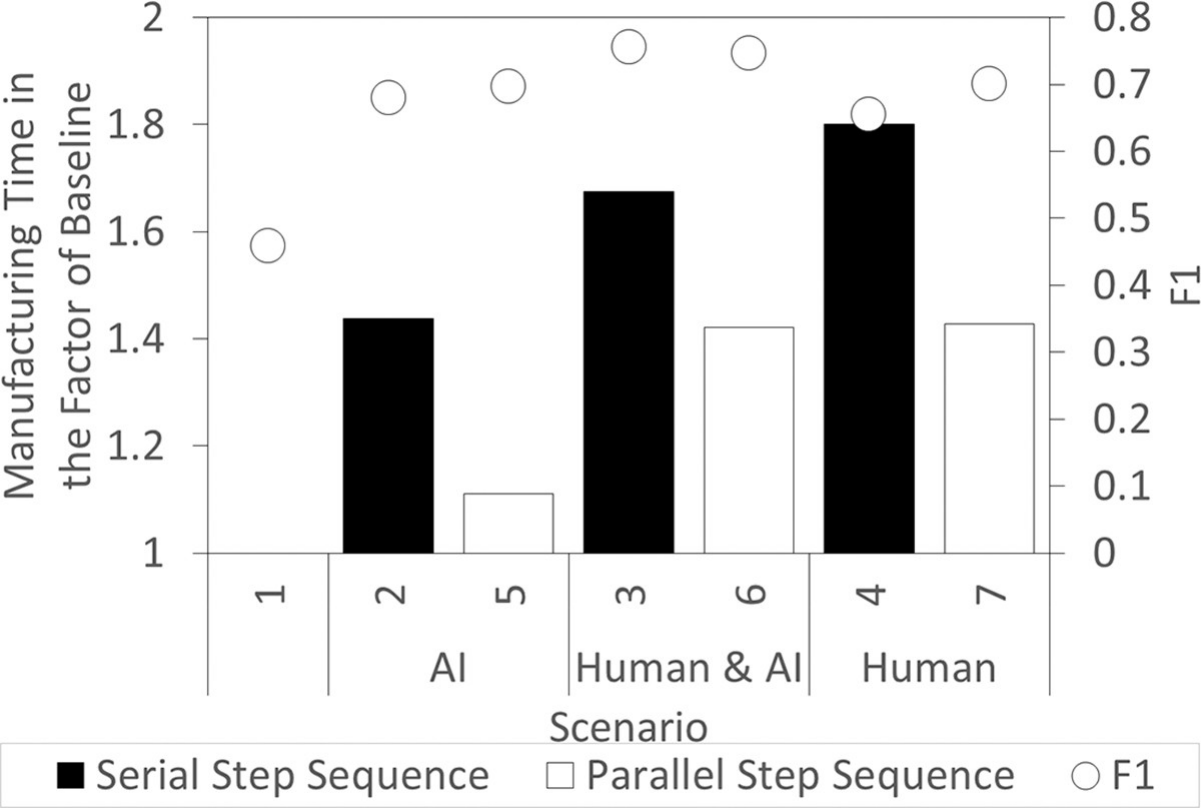
Total production and inspection time to assembly five desktop computers and the F1 score for each scenario with Scenario 1 as the baseline.

Fig 6 shows the total time taken for the production and QC to complete and the F1 score for each scenario. The values are the ratio of time steps taken for each scenario vs Scenario 1 (baseline). Assuming a time step of 1 second, Scenario 1 took an average of approximately 46 minutes (2787 time steps) to complete all the assemblies.

From Fig 6, it can be seen that BCQC took a longer time to complete the five desktop computer assemblies. This is because each workpiece had to be inspected multiple times for each assembly step. In BCQC, at least 50% of the inspectors had to agree on an inspection result. Despite the extra time required, BCQC traded this inefficiency with an increased F1 score in detecting defective workpieces. All scenarios in BCQC achieved above 0.60 F1 scores compared to Scenario 1. A benefit of a high F1 score is the reduction in manpower and material consumption for defective workpieces. Moreover, BCQC provided an alternative for these defective workpieces which will otherwise be scrapped. With early defect detection preventing the irreversible effect of stacked defects, these defective workpieces can be reworked. Besides that, the manufacturing time of all Serial Step Sequence (Scenarios 2, 3, and 4) was longer than all Parallel Step Sequence (Scenarios 5, 6, and 7) with respect to the same type of inspector. This reduction in time could be explained by an effective distribution of workpiece traffic owing to the transparency in the blockchain data.

In this simulation, the effect of BCQC on manufacturing was demonstrated in terms of quality and time. BCQC improved data transparency and promoted better communication. This has enabled the possibility of steps to be executed in parallel. Such parallelism reduced the chances of bottlenecks in the production line and allowed for early defect detection. It was assumed that virtual inspections were performed to achieve a short QC time. Nevertheless, even with the positive effect on quality, the time taken to complete the five workpieces was still longer than the Conventional framework. This was even more apparent when all inspectors were human. Hence, the effect of QDT on the manufacturing time was investigated in the subsequent section.

### Production-independent QC

The focus of this simulation is to observe the effect of BCQC with QDT on manufacturing time. Physical inspections take longer time than virtual inspections and would have a more prominent effect on the manufacturing time. Hence, physical inspections were performed on the workpieces. Each workpiece was transported around for inspection while each workcell inspected one workpiece at a time. Similarly, the workpieces are sent into the QC process one at a time. For example, Workpiece 2 will only be sent for inspection after Workpiece 1 has been fully inspected for all production steps.

**Fig 7:**
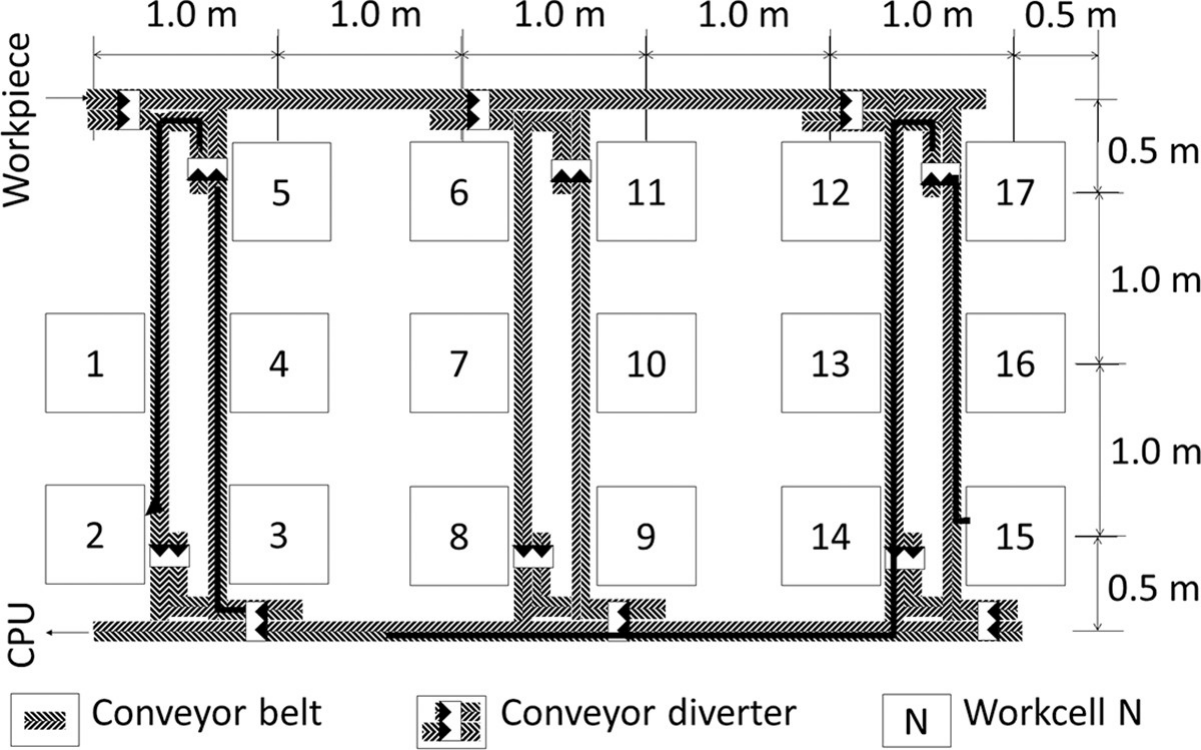
Workpiece transport route from Workcell 15 to Workcell 2 following the direction of the conveyor belt.

The speed of the conveyor belt was set at a constant of 0.05 m/s and the time taken of the conveyor diverter to split the belt was set at a constant of 1 s. Then, the time taken to transport the workpiece was calculated based on the distance between the source and the sink workcells as shown in Fig 7. Taking the longest route as an example; the travel distance between Workcell 15 (source) and Workcell 2 (sink) is about 11 m and goes through three conveyor diverters. This means that it would take about 223 s to transport the workpiece from source to sink. It is assumed that each workcell prioritizes workcells that are within a travelling range of 2 m from itself for the next inspection. If there are no available workcells within the initial travelling range, the range would be gradually increased until a workcell is found. Due to the design of QDT, it may not be necessary for all workcells to inspect the workpiece before reaching a consensus. This approach can reduce unnecessary transportation time which may be a bottleneck during the multiple inspection processes.

To highlight the effect of QDT on time, Scenario 7 was selected, as human inspectors require more time for inspection than AI inspectors. For ease of evaluation, the time taken for QC was normalized by the production time.

**Fig 8:**
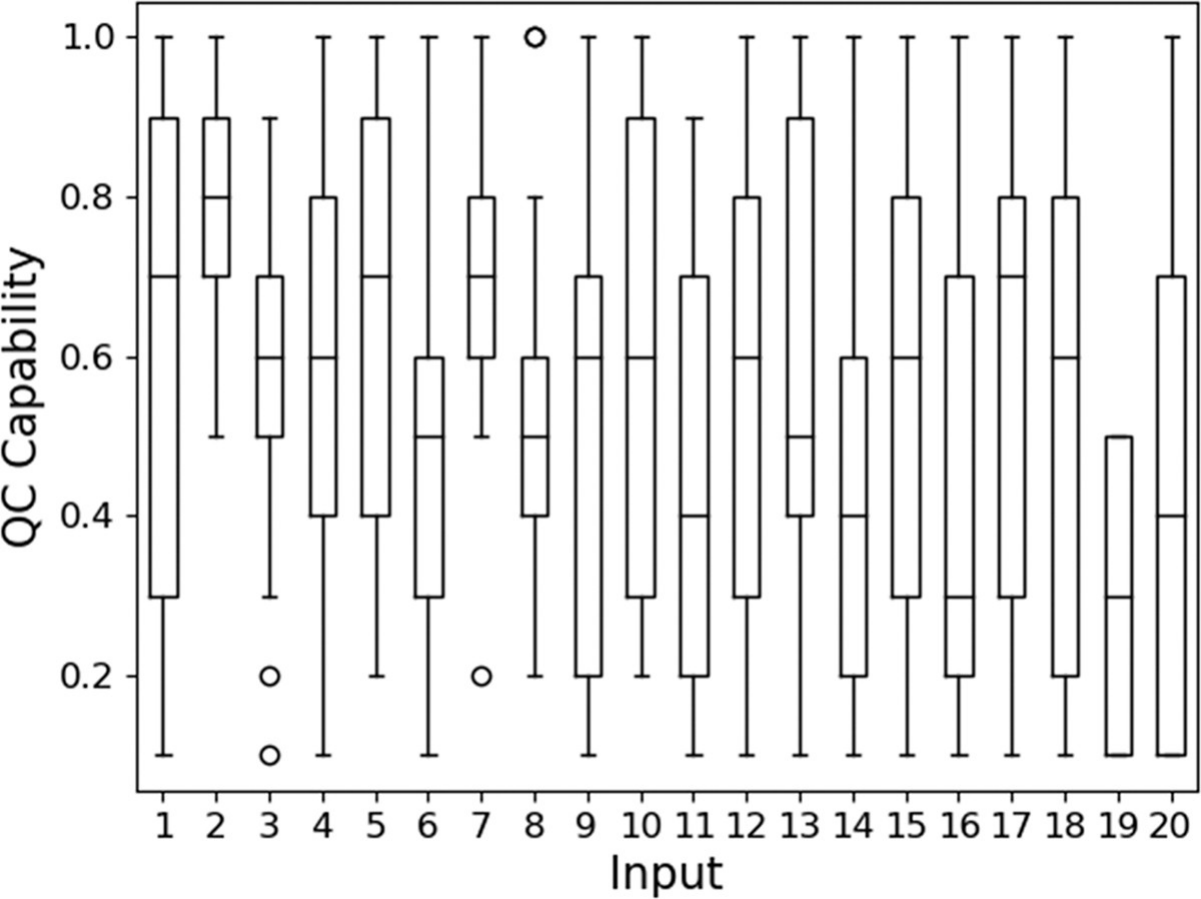
Distribution of QC capability between the scale of 0 to 1 for each input sets.

The actual quality of a workpiece at a particular production step was represented by a percentage. For example, the actual quality of a workpiece could be 70%, where it conforms to 70% of its specifications. On the other hand, each inspection result was generated randomly in a weighted manner. As shown in Fig 8, the capability of an inspector ranged on the scale of 0 to 1, with 0 being the least capable and 1 being the most capable. These capability values were normalized with the actual quality as weights to generate inspection results. A capability of 0.5 indicates that the inspector conforms completely to the 70:30 weighted ratio of the actual quality. Beyond 0.5, this ratio increases, while below 0.5, this ratio decreases.

A total of 20 input sets, with the quality of five workpieces predetermined, were generated for our simulation study. Among the 20 input sets, the inspectors can be highly skilled (very capable), average in capability, or below-average in capability. The distribution of capability of the inspectors in each input set, as shown in Fig 8, is random. This is representative of the various scenarios in a real shopfloor. This includes extreme cases. For example, all the inspectors in Input 2 are highly capable; all the inspectors in Input 19 are highly incapable; the capabilities of inspectors in Input 1 are balanced with a mix of inspectors of different capabilities.

#### QDT with majority consensus

The score for each inspector was set as a constant of 1. The score of an inspection result is given in Eq 2. The minimum allowable nodes are 3. For evaluation, the threshold of the score for consensus was varied from this minimum value to the maximum odd numbers, i.e. 3, 5, 7, 9, 11, 13, 15, and 17. Since the threshold is equal to the number of inspectors agreeing to an inspection result, this mode is termed “majority consensus”. Note that in the previous simulation, a consensus was formed when 51% of the nodes agree on the inspection result. This is equivalent to the threshold value of 9 in this simulation.

$$score_{results}=\sum_{i=1}^{17}scor{e_{qc}}_{i}$$ (2)

**Fig 9:**
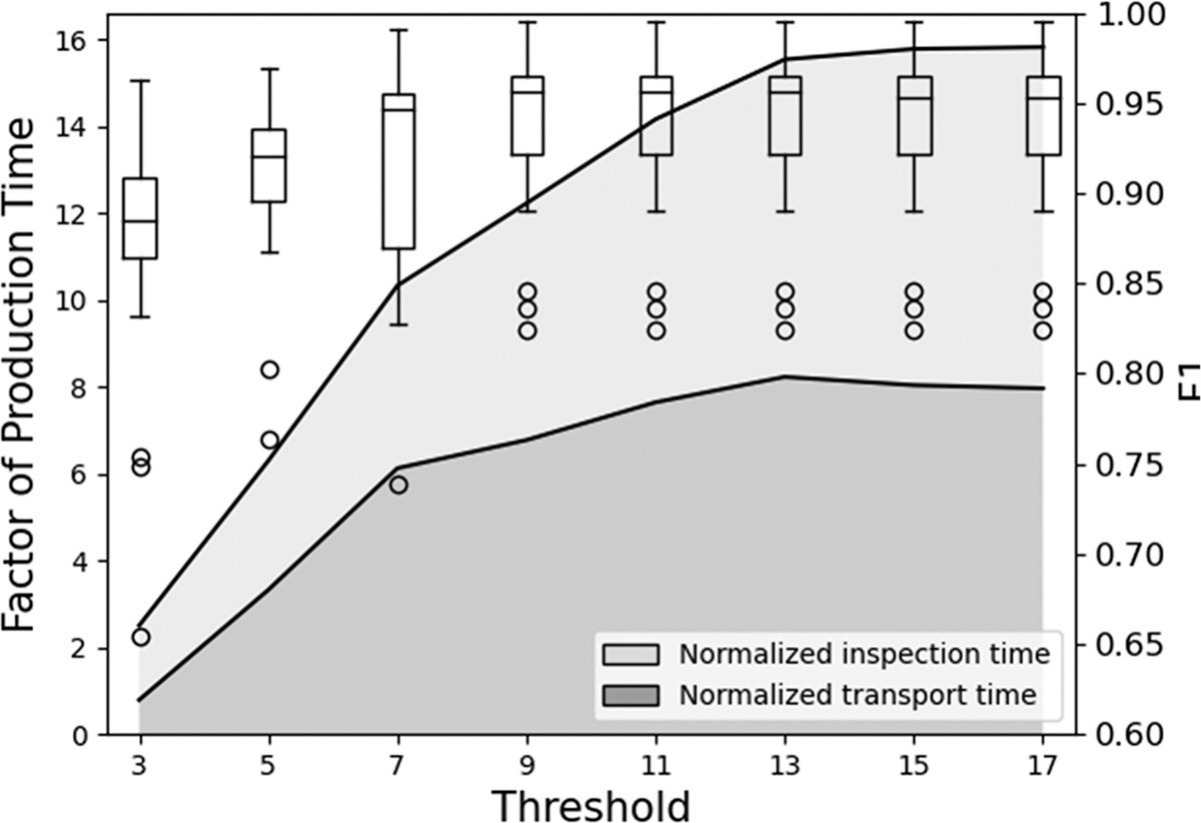
Distribution of F1 score and stacked area of average normalized QC time across results of 20 input sets. The QC time is the sum of transport time and inspection time. Normalization of the QC time is based on a factor of production time of the respective input sets.

The simulation results showed that the program output for each input set across three trials has small variations. Hence, one trial from each input set was compared. Fig 9 shows a generalizable trend for the QC F1 score. F1 score increased with the threshold and plateaued at threshold value = 9, which is beyond 51% of the total inspectors. Additionally, the maximum F1 score was lower than one because the actual quality for some assembly steps of the workpieces was near to 50% “pass” and 50% “fail. This lowers the chances for the consensus results to match the ground truth. Nevertheless, all thresholds resulted in F1 scores of 0.8 and above. Among the input sets, Inputs 2 and 7 had exceptionally high F1 score, i.e. 0.95 and above, starting from threshold value = 3 due to having all inspectors being highly capable.

It is possible to have a lower F1 score in simulations with a higher threshold. This can be observed in Input 3 with threshold values from 5 to 7, Inputs 15 and 18 at threshold 5, and Inputs 4, 6, 11, 12, 14, and 20 at threshold 7. Such cases were caused by having three, four, and six incapable inspectors stationed within the same zone across threshold values of 5, 7, and 9 respectively. Since the next inspector is selected based on distance, a nearby but incapable inspector may be chosen. Hypothetically speaking, if multiple incapable inspectors exist within a region, incapable inspectors may be consecutively selected. Hence, a consensus will be reached even with the wrong inspection results, when majority of the inspectors make similar erroneous judgement. One possible method to mitigate such an issue is to distribute incapable inspectors in different zones. However, this presumes that the actual capabilities of the inspectors are known before they are assigned.

Fig 9 also shows a generalizable trend for QC time. The maximum and minimum standard deviation for the normalized transport time of the five workpieces were 0.34 and 0.02 respectively. The maximum and minimum standard deviation for the normalized inspection time of the whole production were 0.19 and 0.04 respectively. These small values showed the consistency of the results obtained. Although the chosen routes between each inspection were the shortest, the transport time was still longer than the inspection time. This would vary if the speed of transportation increases or the shopfloor map is modified. Nevertheless, these values caused an increase of approximately three times the production time for every increase of two nodes in the consensus threshold.

Although a threshold value of 9 gave the maximum achievable F1 score, the total time required to complete the QC process for five workpieces was 12 times the production time. This means that if the production time without QC is 2 hours, it would require an additional 24 hours to perform the QC process. This would be considered too costly for manufacturers. Thus, there was a need to find a threshold that could achieve an acceptable F1 score with the lowest possible total time to compete in the QC process.

#### QDT with confidence consensus

Another method for score calculation was investigated with the aim to obtain a high F1 score with the lowest possible QC time. In this method, the estimation of capability assigned during input generation was used as the score. Since the capability of each inspector represents its confidence in its inspection result, this mode is termed as “confidence consensus”. The score of an inspection result is given in Eq 3. The threshold of the score for the confidence consensus was set at 0.9, while the minimum allowable nodes was fixed at 3 which was computed from Eq 3. The threshold is the targeted F1 score to be achieved.

$$P(qc_{n}\;capable)=scor{e_{qc}}_{n}$$

$$P(qc_{n}\;incapable)=1–P(qc_{n}\;capable)$$

$$P(all\;qc\;incapable)=P(qc_{1}\;incapable)\times P(qc_{2}\;incapable)\times \ldots \times P(qc\_17\;incapable)$$

P(≥1qccapable)=1–P(allqcincapable)

$$score_{results}=P(\geq 1\;qc\;capable)$$ (3)

**Fig 10:**
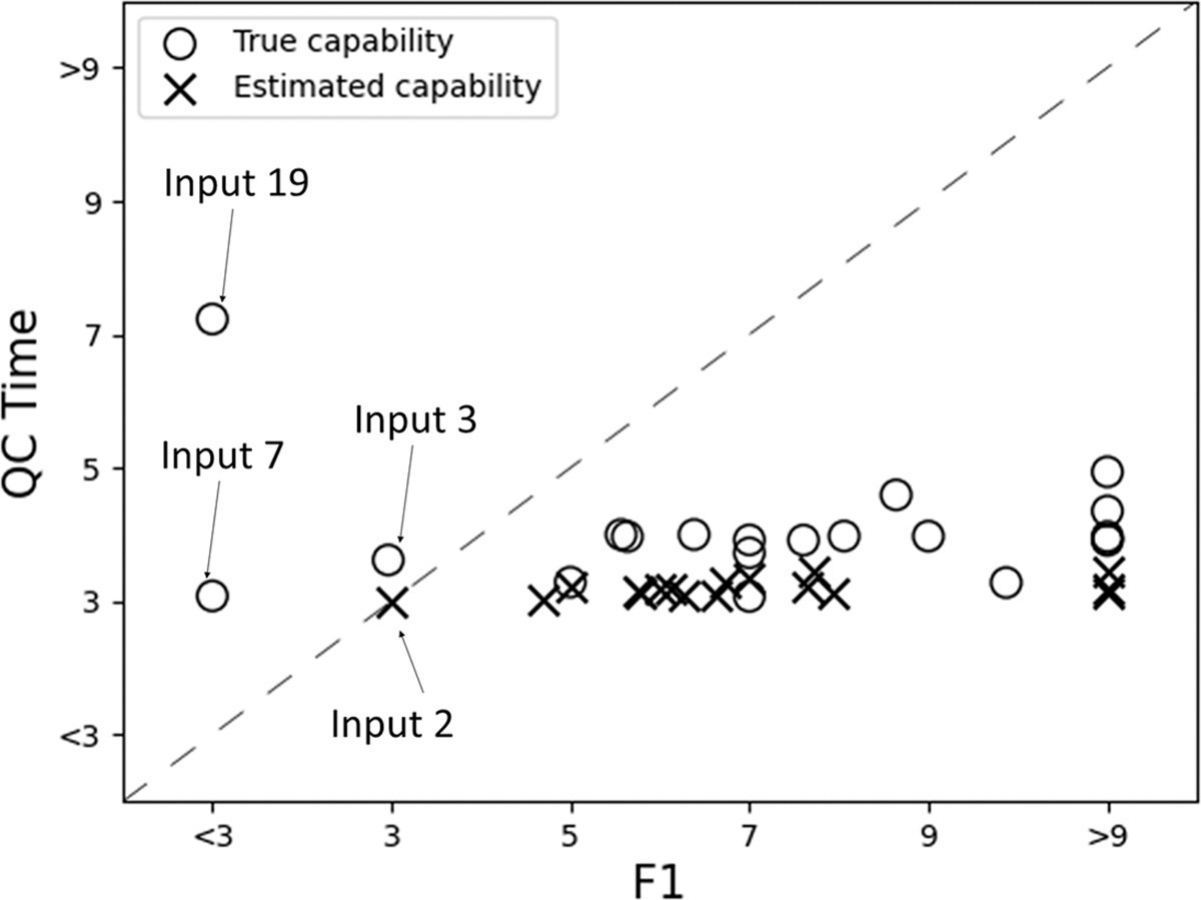
Equivalent threshold of majority consensus for each run in confidence consensus. The normalized QC time (sum of transport time and inspection time) as factor of production time of the respective input sets.

To compare the results between the two methods, this confidence consensus was applied to the same 20 input sets. For each input set, the F1 score of the confidence consensus was compared with the F1 scores of majority consensus across threshold values of 3, 5, 7, and 9 inspectors; the equivalent majority consensus threshold value was recorded. For example, for Input 2, the F1 score of confidence consensus was 0.98 and the F1 score of majority consensus at threshold value = 7 was 0.98. Hence, the equivalent majority consensus threshold value of F1 for this input set was 7. The same comparison was done for QC time across the input sets. Results of such comparisons were charted in Fig 10 and labeled as “true capability”. To obtain a high F1 score with a short QC time, the equivalent threshold for the F1 score would have to be higher than the equivalent threshold for QC time, i.e. the points would have to lay below the dashed line.

From Fig 10, it is observed that all input sets except three (Inputs 3, 7, and 19) showed the aforementioned trends. Results from Input 19 was due to a high number of incapable inspectors. Results for Input 3 were due to three incapable inspectors stationed within the same zone, and hence, the F1 score at threshold value = 3 was higher than the F1 scores across threshold values of 5 and 7. Results for Input 7 were caused by an exceptionally high F1 score in the majority consensus, which was above 0.96, and thus, an F1 score of 0.94 in the confidence consensus was sufficient to achieve consensus. Among these three outliers, only Input 19 with the confidence consensus showed a lower F1 score than the results of Input 19 with the majority consensus. The general trend of the 17 input sets in Fig 10 achieved equivalent threshold value = 5 and below for the QC time. This threshold value = 5 of the majority consensus, as shown in Fig 9, completed the QC process within six times the production time. This proved that the QC time in confidence consensus was shorter than that in majority consensus.

The actual capability of each inspector may not be known in practice. Hence, there is a need to estimate these capability values. In this simulation, this estimation was learned by assigning weights to the inspection results of each inspector. These weights are not the actual capability of the inspectors. Each weight was assigned with a low value at the start of each simulation. This assignment was for determining the actual capability of the inspector. The assigned weights were iteratively increased or decreased to a value that represents the actual capability of the inspector. Note that, to estimate the capability accurately, the inspectors need to inspect workpieces from a wide range of production steps to avoid bias. If an inspector only inspects workpieces that are passed from its neighbours, the estimated capability will be biased. Therefore, the next node to perform quality control should be selected at random and not by the nearest travel distance as is assumed in the non-training simulations. The score for each inspector was initially set at 0.1 so that almost all 17 inspectors will be involved in the first inspection round to achieve the threshold. There was a reward (+0.01) and a penalty (-0.01) given to update the score for every inspection made. These values were arbitrarily chosen. Other optimization methods, such as the gradient descent method, may be applied, but such application is out of the scope of this paper. Since the score was not adjusted based on the actual capability, this could be considered as an unsupervised learning process.

To add a convergence condition to the aforementioned approach during the training period, inspectors had to keep track of the number of times they belong to a QC list, *N*_*qc*_, and the number of times they belong to a winner group, *N*_*winner*_. These counters tracked the history of success, i.e. *N*_*winner*_/*N*_*qc*_. The history of success was reset to 0 for every *N*_*qc*_ = 10. The error in the estimated capability was given by the differences between the history of success and the score. A reward would be given if the inspector belongs to the winner group of that inspection and the error is more than +0.01. A penalty would be given if the inspector is in the QC list but did not belong to the winner group and the error is less than -0.01. The scores were capped between 0 to 1.

**Fig 11:**
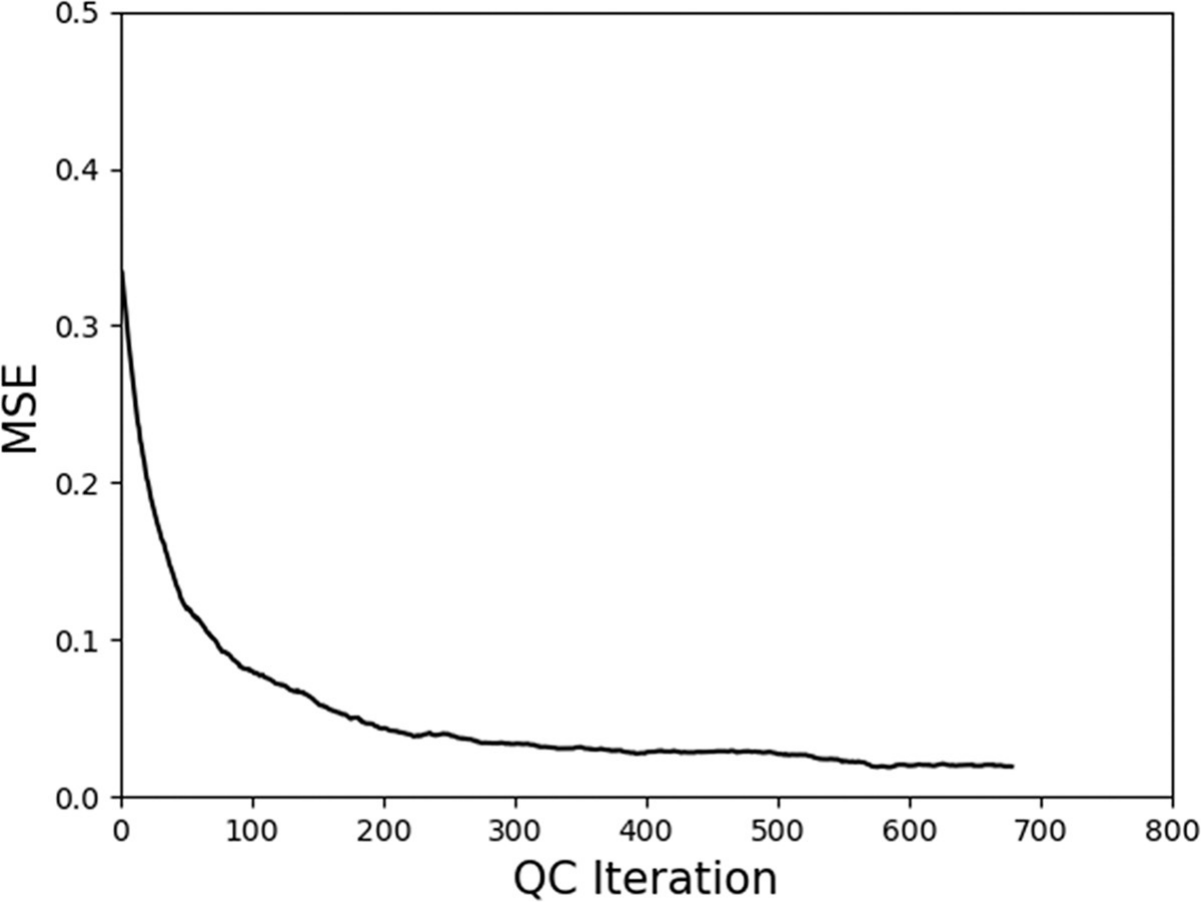
MSE between the estimated capability and the true capability of the trained system using Input 1. All 17 trained input sets showed similar results.

During the simulations, convergence could not be achieved when QDT was tasked to learn with the previously generated 20 input sets. This failure might be due to the short production time needed to assemble five workpieces. To increase this production time, an additional input set for assembling 20 workpieces was generated. These inputs used the same inspectors as in the previous 17 input sets (excluding the outliers: Inputs 3, 7, and 19). Fig 11 shows the mean square error (MSE) between the estimated capability and the true capability of Input 1. MSE from the trained input sets shows a similar trend, and thus, only one example is shown. The curve indicates that the system was learning the capability of the inspector until it converged to a small MSE, a value of less than 0.05.

The estimated capability values were then used as scores for the confidence consensus simulation on the 17 input sets to assemble five workpieces. The results were again compared with the F1 score and QC time in majority consensus, charted onto Fig 10 and labeled as “estimated capability”. The estimated capability values contributed to lower F1 scores and longer QC times. Nevertheless, when compared to QC processes with majority consensus and equivalent F1 scores, the QC times were still shorter. Only Input 2 was an exception. Similar to Input 7 in the previous simulation for QDT in majority consensus, Input 2 was an outlier because of its exceptionally high F1 score in the majority consensus, which was 0.95 and above. This implies that when all inspectors are highly capable, the difference between the majority consensus and the confidence consensus is minimal as a high F1 score would still be achieved.

In this simulation, the QC of each workpiece was carried out independently and the results from the QC did not affect the production step sequence. The proposed QDT with majority consensus showed that the time taken for the QC steadily increased with the number of inspectors. Similarly, the F1 score increased with the number of inspectors involved but plateaued when the number reaches beyond 51% of the total inspectors available. On the other hand, the proposed QDT with confidence consensus could achieve a high F1 score promptly. It was also found that training sessions with QDT could estimate the capability values of each inspector with MSE below 0.05.

### Production-dependent QC with QDT

The results from QDT with confidence consensus can be used to determine the next production step sequence. Hence, the focus of this simulation is to observe the effect of this action on the F1 score and total manufacturing time. Physical workpieces were still transported from workcell to workcell for physical inspection. The difference from the production-independent simulation is that inspections of all workpieces were performed immediately after each respective production step. The actual quality of the workpiece for each step was generated randomly. The values of actual quality were used in the generation of the confusion matrix during data analysis.

**Fig 12:**
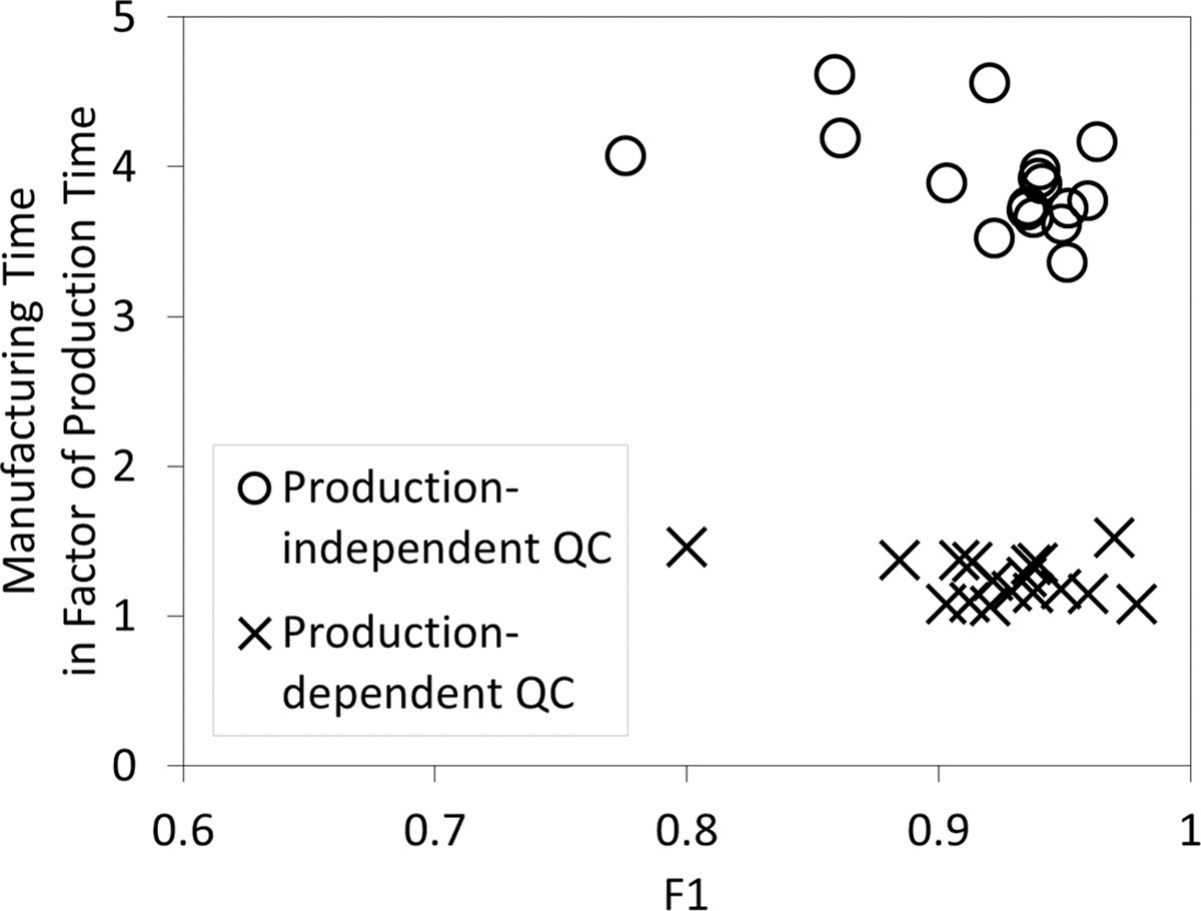
Scatter plot of normalized manufacturing time against the F1 score for all 17 inputs to manufacture five workpieces.

The actual capabilities from the 17 inputs in Fig 8 were input as capability values to generate inspection results, while the estimated capability values from the aforementioned simulations were input as scores to perform score calculations in QDT. The inputs for the production step sequence were obtained on the spot based on the inspection results that are consolidated in the achieved consensus. The results from this simulation was compared with the results from the previous simulation for QDT in confidence consensus in Fig 12. The manufacturing time needed to complete five workpieces was less than two times the production time. Note that, there is a difference in the nature of manufacturing time in this simulation and in the production-independent QC simulation. Here, production time, workpiece transport time, and inspection time overlaps, whereas in production-independent QC simulation, these time variables are mutually exclusive. On the other hand, the F1 score remained within the same range when compared with the production-independent QC simulation.

In this simulation, the QC of each workpiece affected the production step sequence based on inspection results and availability of the workcells. The shorter manufacturing time taken to complete five workpieces greatly reduced the total manufacturing time taken when the QC processes overlapped with the production processes. Nevertheless, the workpiece quality that is represented by the F1 score was maintained.

### Data security issues

In a CPS, various layers are susceptible to security attacks due to the openness of a CPS network. The possible vulnerabilities to be discussed in this paper are attacks to sensors, communication channels, and end devices.

Inspection results might be tampered with while being sent from one node to another. Besides validating the hashes of the chain, the inspection results that are mined into a block would have to be validated. These validations occur at each node before the block is accepted into the chain. While the validations are carried out at each node, hijacked data at nodes or communication channels would have a higher chance to be detected.

The inspection results, which are stored in a decentralized manner when forming the blockchain, helps detect anomalies in sensors, communication channels, and nodes. Each node is only authorized to transfer data related to its own inspection results to another node. If a node begins to show anomalies in the middle of the QC process, the data from earlier transfers would be different from that of later transfers. If a node is attacked, not all data will be lost as other nodes would still have a copy of the data.

Sensors might malfunction or an agent might miss a defect that results in mistakes in an inspection. While this distributed peer-to-peer supervision provides redundancy, the requisite workpiece transportation is usually time consuming and unproductive. The proposed QDT balances the inspection redundancy and workpiece transportation time to ensure product quality at the lowest possible time. The data sharing between nodes in a CPS makes it possible to find the nearest available node to perform an inspection.

## Conclusions

In this paper, we proposed an original framework, Blockchain Quality Control for the application of blockchain in a decentralized Cyber-Physical-System-based workpiece quality control system. We also proposed a consensus algorithm, Quality Defect Tolerance, designed specifically for quality control system. Existing blockchain focuses on securing transaction data from a single source and selecting a node as the block creator. Our approach differs by enabling blockchain to select the most trustworthy data from numerous data sources. During quality control processes, each inspector may have different inspection results on the same workpiece. Our approach provides the feature of consolidating differing inspection results in Quality Defect Tolerance and storing the results into a secured blockchain with Blockchain Quality Control. Extensive computer simulation has demonstrated the effectiveness of the Blockchain Quality Control and Quality Defect Tolerance, advancing the state-of-the-art of blockchain in manufacturing. Blockchain technology could enhance manufacturing accuracy, especially for mass personalization production.

Multiple workpiece inspections in the form of peer-to-peer supervision improve product quality. In the simulations with Quality Defect Tolerance in majority consensus, the quality control quality and quality control time increased with the number of inspectors. Still, the quality control quality reached its maximum when >51% of nodes are used as the consensus threshold. With Quality Defect Tolerance in confidence consensus, this maximum quality could be achieved within a shorter time as compared to Quality Defect Tolerance in majority consensus. The manufacturing time was less than two times the production time when Blockchain Quality Control with Quality Defect Tolerance in confidence consensus was implemented. Besides that, existing literature has shown that blockchain as a distributed ledger may strengthen data security [22], provide data immutability [1], and improve data transparency [3] for real-time updates on the assembly process.

However, there are limitations to this study. Similar to Proof-of-Work [29], an attack on a majority of the nodes, i.e. 51%, could tamper with the inspection results. This attack would not be easily detected because consensus can be achieved as long as the score of the results exceeds the threshold. To overcome this, the cost of the attack has to exceed the benefits of such tampering. Practically, there is usually a timeframe of vulnerability after the consensus and before the conflict resolution. During conflict resolution, if the quality control list and the winner group are attacked, the corrupted blockchain can be spread to all the nodes. Uncorrupted data may be lost if the data are not backed up periodically on a separate storage system. Besides that, repeated erroneous inspections that occur within an area reduces the effectiveness of Quality Defect Tolerance. Such repetition increases the likelihood of accepting the erroneous results. Referring to the aforementioned simulations on confidence consensus, if the erroneous results are accepted, these inspectors will receive an increment in their weights and will mistakenly be estimated as having higher capabilities. Similarly if undetected, the confidence of these less skilled inspectors could increase and affect the future consensus, even when they were no longer clustered within the same zone.

Nevertheless, the application of blockchain in quality control is still in its infancy that requires further explorations on its potentials, such as optimum node configurations and artificial intelligent-based virtual inspection methods. Our next step includes applying smart contracts to govern the rules of Quality Defect Tolerance. The smart contract may decide on the consensus threshold and learn the capability of inspectors autonomously. We also intend to integrate the Blockchain Quality Control with a process controller that consists of a cognitive engine. The blockchain may be passed to the process controller to conduct dynamic scheduling.
